# Effect of Nonsurgical Periodontal Treatment on Salivary and Plasma Superoxide Dismutase Levels of Patients Suffering from Periodontitis

**DOI:** 10.3390/jcm12206688

**Published:** 2023-10-23

**Authors:** Tanja Veljovic, Milanko Djuric, Jelena Mirnic, Ivana Gusic, Aleksandra Maletin, Stojan Ivic, Marija Stojilkovic, Snezana Brkic

**Affiliations:** 1 Department of Dental Medicine, Faculty of Medicine, University of Novi Sad, 21000 Novi Sad, Serbia; milanko.djuric@mf.uns.ac.rs (M.D.); jelena.mirnic@mf.uns.ac.rs (J.M.); ivana.gusic@mf.uns.ac.rs (I.G.); aleksandra.maletin@mf.uns.ac.rs (A.M.); stojan.ivic@mf.uns.ac.rs (S.I.); marija.stojilkovic@mf.uns.ac.rs (M.S.); snezana.brkic@mf.uns.ac.rs (S.B.); 2 Dentistry Clinic of Vojvodina, Department of Dental Medicine, Faculty of Medicine, University of Novi Sad, 21000 Novi Sad, Serbia; 3 Clinic for Infectious Diseases, Clinical Centre of Vojvodina, Faculty of Medicine, University of Novi Sad, 21000 Novi Sad, Serbia

**Keywords:** superoxide dismutase, oxidative stress, periodontal disease, plasma, treatment

## Abstract

Antioxidant capacity is frequently measured by evaluating superoxide dismutase (SOD) concentration in body fluids. The aim of this study was to compare SOD concentrations in the saliva and plasma of patients with periodontitis to those measured in a group of patients with healthy periodontium, as well as to evaluate the influence of nonsurgical periodontal therapy on salivary and plasma SOD in periodontitis patients. For this purpose, 40 systemically healthy patients aged 30–70 years who had at least 20 teeth were recruited, 20 of whom had periodontitis, and 20 served as healthy periodontitis-free controls. In all participants, periodontal status was assessed via the plaque index (PI), gingival index (GI), papilla bleeding index (PIB), probing depth (PD), and clinical attachment level (CAL), and the SOD concentration in both saliva and plasma was determined by conducting a commercial immunoenzymatic ELISA test. In periodontitis patients, periodontal indices and saliva and blood samples were taken at the beginning of the study, as well as 3 months after periodontal therapy, while in the control group, these data were gathered at the beginning of the study only. SOD values in the saliva of patients with periodontitis (0.244 U/µL) were statistically significantly higher compared with patients with healthy periodontium (0.017 U/µL). Moreover, periodontal therapy led to a statistically significant decrease in this marker in the saliva of patients with periodontitis (*p* = 0.023), which was comparable with that measured in the control group. On the other hand, no statistically significant differences were noted in plasma SOD values either between the two groups or at follow-up compared with baseline in the group with periodontitis. These findings suggest that the elevated salivary SOD in patients with periodontal disease may represent a mechanism of tissue protection against oxidative stress that occurs in response to periodontal disease.

## 1. Introduction

A discrepancy in the production of free oxygen species (ROS) and antioxidant protection leads to the occurrence of oxidative stress. Today, oxidative stress is recognized as an important factor in the development of serious pathological changes and systemic diseases, such as atherosclerosis, diabetes, rheumatoid arthritis, neurodegenerative diseases, brain and heart infarction, and cancer [1,2,3,4,5,6]. More recent evidence also supports the hypothesis that oxidative stress can be involved in the etiopathogenesis of periodontal disease [7,8].

Research shows that, in addition to periodontitis, oxidative stress in the oral cavity is also associated with other local factors and diseases, such as oral lichen planus, oral cancer, leukoplakia, recurrent aphthous stomatitis, caries, and periapical lesions [9,10,11,12,13,14,15]. The extant literature further suggests that different treatment protocols, such as nonsurgical periodontal therapy, laser and photoactivation therapy, endodontic treatment, and tooth extraction, impact the value of oxidative stress markers [13,16,17,18].

Superoxide dismutase (SOD) is considered one of the main antioxidants, which is why it has been extensively studied in patients with periodontitis. As superoxide dismutase is present in all tissues and is also active in body fluids (including blood plasma) [19], changes in its activity have been identified in a number of diseases [20,21,22]. Accordingly, in the periodontal ligament, SOD may represent an important defense mechanism of the gingival tissue against superoxide release [23]. This assertion is supported by the evidence presented by Na et al. indicating significantly increased SOD levels in inflamed gingival tissue compared with healthy gingiva, suggesting the presence of a positive link between SOD activity and the degree of inflammation [24]. On the other hand, periodontal therapy has been shown to be beneficial for the reduction of this marker in the saliva of patients with periodontitis [25,26,27]. However, in several studies, salivary SOD values were lower in patients with periodontitis compared with controls and increased upon periodontal therapy completion [28,29].

Periodontitis has gained prominence in recent years due to its influence on systemic health and the occurrence of some systemic diseases. In addition to the infectious and inflammatory component of periodontal lesions, oxidative stress as a possible link between periodontitis and systemic diseases has received growing attention from both researchers and healthcare practitioners. Namely, recent studies show that periodontitis is not only accompanied by an imbalance between free radical production and antioxidant protection in the oral cavity but that reactive forms of oxygen produced during periodontitis diffuse into the blood, leading to the oxidation of blood biomolecules and the emergence of circulatory oxidative stress and diseases affecting distant organs [30].

However, studies in which SOD values in the blood of patients with periodontitis were assessed have yielded contradictory results; in some cases, it was lower, while in others, it was higher compared with healthy patients [25,31,32,33,34,35,36]. Therefore, there is a need for further research into this topic.

Against this background, the aim of the present study was to compare the salivary and plasma SOD values of patients with periodontitis with those measured in patients with healthy periodontium, as well as to examine the influence of nonsurgical periodontal therapy provided to patients with periodontitis on SOD levels in both saliva and plasma.

## 2. Materials and Methods

### 2.1. Research Participants

To meet the aforementioned study objectives, 40 patients aged 30–70 who had at least 20 teeth and were systemically healthy were recruited for this research. Patients who (1) had a systemic disease that may affect the periodontium, (2) had periodontal treatment in the previous six months, (3) had received antibiotics or anti-inflammatory drugs in the last three months, (4) had taken vitamin supplements, (5) were smokers or (5) were pregnant or lactating were excluded.

Prior to commencing the research, approval was obtained from the local ethics committee, and all prospective study participants were informed about the study aims and protocols, as well as the nature of their involvement, both verbally and in writing. Only those who signed an informed consent form participated in the study interventions, which fully conformed to the Declaration of Helsinki.

The periodontitis group included patients with a minimum of two sites at which clinical attachment level (CAL) and probing depth (PD) of ≥3 mm and ≥4 mm at different teeth were identified, respectively, or PD ≥ 5 mm was noted at one site [37]. The control group consisted of patients with healthy periodontium who maintained good oral hygiene.

Upon examination, 20 individuals (13 females and 7 males; mean age = 52.1 years) were confirmed to meet the aforementioned criteria and were placed into the periodontitis group, while the remaining 20 patients (12 females and 8 males; mean age = 50.6 years) formed the control group. According to the power analysis, this sample size was adequate, as 95% power and 95% significance level could be attained with only 18 patients.

### 2.2. Periodontal Examination

At the beginning of the study, all patients underwent a clinical assessment, whereby the periodontal status of all their present teeth (with the exception of the third molars) was assessed by measuring plaque index (PI) [38], gingival index (GI) [39], papilla bleeding index (PIB) [40], probing depth (PD), and clinical attachment level (CAL).

These indices were employed in prior research conducted by our group, given that they best reflect periodontium status [16,41,42]. To obtain their values, Michigan ‘O’ probe with William’s markings was used by the same periodontist to perform measurements on the mesiobuccal, distobuccal, midbuccal, and midlingual tooth surfaces.

### 2.3. Sample Collection and Preparation

At the start of the study, all patients provided mixed unstimulated saliva samples, which were used to establish the concentration of SOD at baseline. They were instructed to abstain from all food and beverages prior to their morning appointment. The obtained samples were immediately transported to the laboratory, where they were centrifuged at 3000× *g* for 10 min at room temperature, and the subsequently isolated supernatant was stored at −80 °C. During the same initial appointment, the patients provided finger-prick blood samples, which were collected in EDTA-coated tubes (Kabe Labotechnik, Nümbrecht-Elsenroth, Germany). Upon their arrival at the laboratory, the blood samples were centrifuged at 3000× *g* for 10 min to separate plasma, which was stored at −80 °C.

### 2.4. Laboratory Analysis—SOD Determination

The SOD concentration in both saliva and plasma was determined by conducting a commercial immunoenzymatic ELISA test (Cell Biolabs’ OxiSelectTM, San Diego, CA, USA), according to the procedure prescribed by the manufacturer using the spectrophotometric method, whereby the standard curve (ranging from 5 U/μL to 0.61 mU/μL) was adopted to quantitatively determine the results.

### 2.5. Periodontal Therapy

The 20 patients in whom periodontitis was confirmed (the periodontitis group) underwent nonsurgical periodontal treatment consisting of root planing with Gracey curettes and scaling with ultrasonic scalers (Mini Piezon, Electro-Medical Systems, Nyon, Switzerland) without the use of antiseptics or antibiotics. Depending on the severity of periodontitis, 1–2 visits were needed to complete the planned therapy, at the end of which patients were provided detailed instructions on the correct oral hygiene maintenance protocol they should follow daily at home.

### 2.6. Follow-Up

The procedures adopted to collect the blood and saliva samples at the start of the study were repeated three months later for the periodontitis group only and were used to assess the therapy outcome.

### 2.7. Statistical Analysis

The gathered data were subjected to statistical analyses performed using the SPSS Statistics 20 for Windows (SPSS, Chicago, IL, USA) commercial software. All values were presented as mean ± SD, and *p* < 0.05 was considered statistically significant. While pairwise relationships between observed attribute characteristics (gender) were assessed using Pearson χ^2^ test, student’s *t*-test was applied to patient characteristics that were represented numerically, allowing the mean values of age, number of teeth present, periodontal indices, index levels before and after the treatment, SOD level in the two groups, and the SOD marker values before and after treatment to be compared. Spearman’s rank correlation coefficient was calculated to determine the presence/absence of correlation between periodontal clinical parameters and salivary and plasma SOD markers. On the other hand, the Mann–Whitney test was employed when comparing the mean values of clinical parameters and SOD in the periodontal and the control group at baseline, as well as for determining the treatment success in the periodontal group. For this purpose, pre–post-treatment comparison was also performed via the Wilcoxon test.

## 3. Results

As shown in Table 1, at the beginning of the study, patients placed in the periodontitis group had statistically significantly higher values of all examined clinical parameters compared with the controls. Three months after completing the therapy, these patients also exhibited significant changes in PI, GI, PBI, PD, and CAL values. Moreover, a significant reduction in PD was observed upon therapy completion at sites with moderate (5 mm ≥ PD < 7 mm) as well as deep (PD ≥ 7 mm) periodontal pockets (Table 2).

At baseline, patients in the periodontitis group had statistically significantly higher salivary SOD values compared with the controls. A statistically significant decrease in this marker was noted 3 months after therapy completion. Compared with those with healthy periodontium, patients with periodontitis also had higher plasma SOD values, but this difference was not statistically significant. Periodontal therapy led to a minimal decrease in the value of this marker (Table 3).

**Figure 1:**
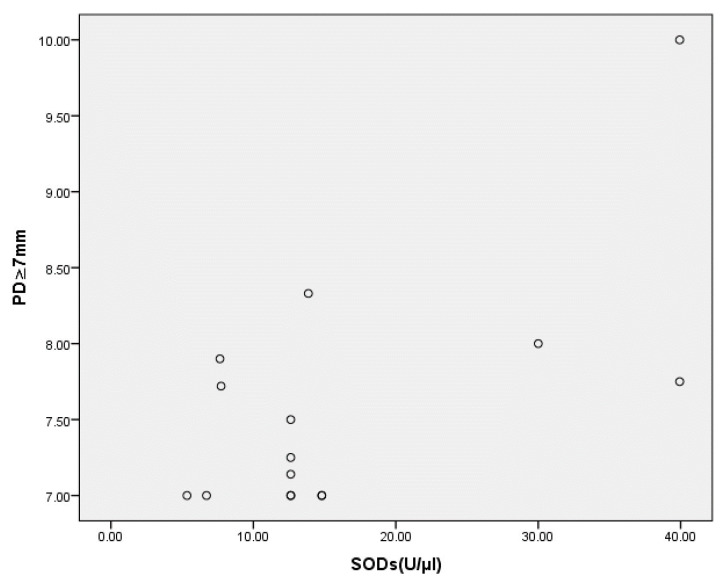
Correlation between deep pocket depth (PD ≥ 7 mm) and SOD level in the saliva of patients assigned to the periodontitis group at baseline. Statistically significant at *p* < 0.01; PD ≥ 7 mm, deep pocket; SODs, superoxide dismutase value in saliva.

**Figure 2:**
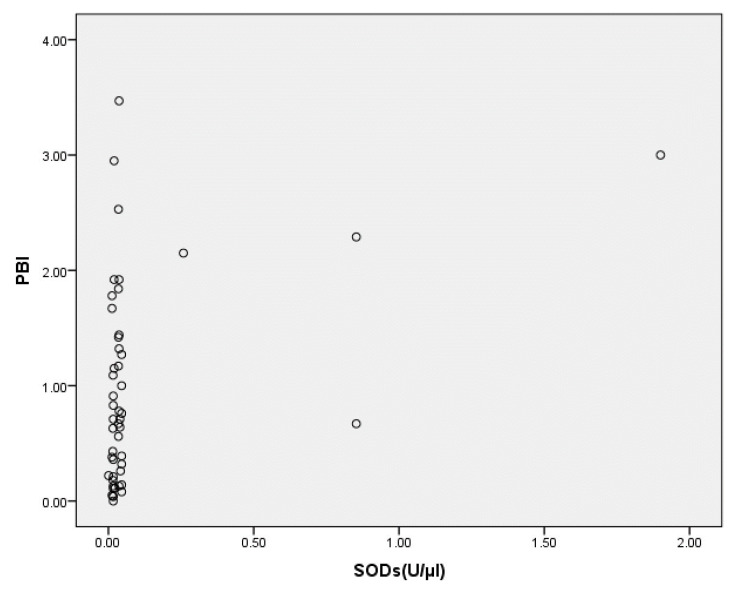
Correlation between PBI and SOD levels in the saliva of patients assigned to the periodontitis group at baseline. PBI, papilla bleeding index; SODs, superoxide dismutase level in saliva.

As can be seen from the results reported in Table 4, salivary SOD was positively correlated with the presence of deep periodontal pockets (i.e., those with PD ≥ 7 mm, *p* = 0.008) (Figure 1), while the correlation with PBI was close to statistical significance (*p* = 0.056) (Figure 2).

## 4. Discussion

Antioxidants are defined as substances that, in small concentrations, have the ability to postpone or inhibit the oxidation of other substances through the mechanism of competition. According to the nature and mode of action, antioxidants can be divided into enzymatic (superoxide dismutase, catalase, glutathione reductase, glutathione S-transferase, etc.) and nonenzymatic (glutathione, vitamins A, C, and E, albumins, ceruloplasmin, transferrin, bilirubin, uric acid, etc.).

Antioxidant capacity is frequently measured by evaluating superoxide dismutase (SOD) concentrations in body fluids. However, changes in SOD values are not interpreted uniformly in extant research, as some authors consider high values of this enzyme as a sign of good antioxidant defense, while a low SOD concentration is viewed as indicative of reduced antioxidant capacity and oxidative stress [43,44]. According to Selvam et al. [43], reduced SOD activity suggests lower capacity to neutralize superoxide anions, which are consequently free to react with hydrogen (and create hydrogen peroxide) or with hydrogen peroxide (to form a hydroxyl radical). When unabated, superoxide sets off a chain reaction whereby different reactive oxygen species are generated, giving rise to all associated adverse effects. Therefore, higher SOD activity seems to impart greater antioxidant protection. Yet, these arguments are countered by several studies in which elevated SOD activity is interpreted as a sign of oxidative stress [45,46,47].

Similar contradictions are evident in the findings obtained in studies focusing specifically on SOD activity in patients with periodontal disease [30,35,48]. Even the investigations conducted by the same researchers have yielded inconsistent results, as exemplified by Akalin et al. [49], who in 2005 reported higher SOD values in patients with periodontitis, only to report lower values in these patients compared with healthy subjects a few years later [50]. The authors attributed these discrepancies to the differences in methodology, the way the samples were stored, the number of patients, as well as the disease activity, and the units in which SOD concentration was expressed. Similarly, Ellis et al. [29] reported a significant and progressive decrease in SOD activity with increasing periodontal pocket depth, while Canacki et al. [51] noted lower SOD activity in the gingival fluid of pregnant women suffering from periodontitis compared with healthy controls. In contrast, Novakovic et al., recorded higher SOD values in patients with evidence of periodontal destruction compared with subjects with healthy periodontium [26]. As a part of their study, Wei et al. [25] examined SOD activity in the saliva and gingival fluid of 48 individuals suffering from chronic periodontitis and compared it with the findings obtained for 35 persons with clinically healthy periodontium. These authors obtained significantly higher SOD values in both tested substrates in the periodontitis group compared with controls.

These results concur with those obtained in the present study, where the SOD values in the saliva of patients with periodontitis (0.244 U/μL) were statistically significantly higher than in patients with healthy periodontium (0.017 U/μL). Accordingly, we posit that increased SOD activity represents an important defense mechanism against inflammatory processes in the periodontium. Namely, during inflammation, bacterial lipopolysaccharides stimulate the release of O_2_ from tissue fibroblasts and polymorphonuclear leukocytes [52]. Due to the increased O_2_ production, SOD generation and activity in the periodontium increase to restore the balance between oxidative stress and antioxidant protection.

This view is supported by the findings reported by Akalin et al. based on a comparison of SOD concentrations in gingival tissue and gingival fluid. As these authors found much higher values of this marker in gingival tissue, they concluded that it is a more reliable medium for testing SOD activity than extracellular fluids where its concentration is extremely low [53]. Our research, however, shows that SOD was detectable in the saliva of all patients. Accordingly, as saliva collection and analysis are much simpler and more cost-effective, these methods can be used to determine the SOD concentration in patients with periodontitis as well as in healthy subjects.

Upon analyzing the influence of certain periodontal parameters on the SOD concentration in the saliva of patients with periodontitis, we also established that inflammation of the gingiva and the destruction of deeper periodontal tissues most likely contributed to its higher value in these patients. Namely, the positive correlation between PBI and SOD was close to statistical significance (*p* = 0.056), while the correlation between this marker and the presence of deep periodontal pockets (PD ≥ 7 mm) was statistically significant. A positive correlation between SOD and gingival inflammation was also reported by Novakovic et al. [26], who attributed this finding to the increased need for SOD in order to protect the gingiva during inflammatory processes.

One of the goals of our research was to determine whether the nonsurgical periodontal therapy provided to patients with periodontitis influenced their salivary and plasma SOD levels. We noted a statistically significant decrease in SOD values in the saliva of our patients 3 months after therapy completion (0.041 U/μL) compared with baseline (0.244 U/μL). These results are comparable to the findings reported by Wei et al. [25], who measured 216.4 U/mg protein at the beginning of the study and 169.8 U/mg protein upon completion. Novaković et al. [26] also recorded statistically significantly lower SOD values after therapy in periodontal patients, but these observations were countered by Karim et al. [28], who obtained higher SOD values after periodontal therapy, as well as by Kim et al. [54], who reported a decrease in SOD values 1 month after therapy followed by an increase after 3 months. These authors attributed these results to the elimination of inflammation of the periodontium and a reduction in oxidative stress, as well as the consequent restoration and increase in antioxidant capacity.

Conversely, we opine that periodontitis, as a chronic disease that lasts for years, leads to the adaptation of the body to constant irritation by free radicals and a compensatory increase in the activity of the most important antioxidant enzyme. According to this perspective, the higher initial SOD value in subjects with periodontitis is actually a compensation due to the constant presence of reactive oxygen species and inflammation. As periodontal therapy reduces the inflammation, the activity of this enzyme subsequently normalizes. This explanation is in line with the findings obtained by Sukhtankar et al. [27], who found that periodontal therapy led to a significant reduction in the gingival SOD, which was comparable to that measured in healthy patients. The authors associated the obtained results with a significant reduction in gingival inflammation after the therapy.

While the causal link between systemic diseases and periodontal status has long been established, the possible impact of periodontal disease on systemic health has recently gained considerable attention, given the growing evidence pointing to the negative correlation between periodontitis and systemic total antioxidant capacity (TAOC). Namely, in patients with periodontitis, the level of gingival and systemic TAOC was found to be lower than in healthy subjects [55]. Moreover, Brock et al. posited that reduced antioxidant capacity in the blood of patients with periodontal disease could be a risk factor for cardiovascular diseases [55].

Accordingly, as a part of the present study, SOD concentrations in the plasma of patients with periodontitis were compared with that in healthy controls, and the effect of periodontal therapy on the levels of this marker in plasma was assessed. Similar investigations were conducted by Kale et al. [56] and Wei et al. [25], who reported a statistically significant increase in plasma SOD concentrations in patients with periodontal disease, which declined following periodontal therapy in the cohort examined by Wei et al. [25]. On the other hand, Sudhakar et al. noted an increase in plasma SOD upon therapy completion, which was statistically significant in the group that also took vitamin E supplements [57].

Unlike these authors, as a part of our study, at baseline, we obtained slightly elevated SOD concentrations in the plasma of patients with periodontitis (1.307 U/μL) compared with healthy subjects (1.112 U/μL). Moreover, the periodontitis patients had comparable plasma SOD values before (1.307 U/μL) and after therapy (1.223 U/μL). It is likely that these discrepancies arise not only from the methodological differences but also from the extent of the periodontal disease in the group that underwent therapy. Namely, our patients had a significantly lower degree of periodontium destruction than the patients who were the focus of research conducted by Kale et al. [56] and Wei et al. [25]. This difference, in our opinion, could have led to significantly higher SOD values in the aforementioned studies because these authors emphasized the important influence of periodontal pocket depth on plasma SOD values.

As with any study of this type, this research is not without its limitations, which should be considered when interpreting the findings reported here. The main limitation of this study stems from the reliance on a single oxidative stress marker, as the inclusion of other markers in the analyses would have certainly yielded more informative findings. Its further limitation arises from a relatively small sample size, leading to small treatment and control groups. Sample selection can also be considered a limitation, as it resulted in a relatively cohesive treatment group. Thus, it would be interesting to examine SOD levels in patients with more severe periodontitis, given that, according to the available research results, the value of this marker is related to the degree of damage to the periodontal tissue, suggesting that different results would likely be obtained in the blood.

## 5. Conclusions

The results obtained as a part of the present study indicate that SOD values in the saliva of patients with periodontitis were statistically significantly higher than those measured in controls, who had healthy periodontium. We also found that the SOD value was most affected by gingival inflammation and the destruction of deeper periodontal tissues. In addition, upon completion of periodontal therapy, a statistically significant decrease in this marker in the saliva of patients with periodontitis was noted, whereas plasma SOD values remained comparable with those measured at baseline and were similar to those measured in controls. Accordingly, we posit that the elevated salivary SOD in patients with periodontal disease may represent a mechanism of tissue protection against oxidative stress induced by periodontal disease.

## Figures and Tables

**Table 1:** Clinical periodontal parameters in the periodontitis and the control group at baseline and 3 months after the periodontitis group completed periodontal therapy (mean ± SD).

	Periodontitis Group at Baseline	Control Group	Periodontitis Group after Therapy Completion	^a^ *p*-Value	^b^ *p*-Value	^c^ *p*-Value
PI	0.943 ± 0.433	0.324 ± 0.318	0.405 ± 0.244	0.000 ***	0.000 ***	0.259
GI	1.012 ± 0.676	0.278 ± 0.441	0.340 ± 0.377	0.002 **	0.000 ***	0.395
PBI	1.519 ± 0.787	0.339 ± 0.463	0.770 ± 0.411	0.000 ***	0.000 ***	0.001 **
PD (mm)	3.130 ± 0.550	1.465 ± 0.179	2.574 ± 0.442	0.000 ***	0.000 ***	0.000 ***
CAL (mm)	2.575 ± 1.157	0.369 ± 0.668	1.633 ± 0.938	0.000 ***	0.000 ***	0.000 ***

PI, plaque index; GI, gingival index; PBI, papilla bleeding index; PD, probing depth; CAL, clinical attachment level. ^a^ *p*-value relates to the differences between the periodontitis and the control (periodontitis-free) group at baseline; ^b^ *p*-value relates to the differences between the pre- and post-treatment values obtained in the periodontitis group; ^c^ *p*-value relates to the differences between the control (periodontitis-free) and the periodontitis group 3 months after the latter completed periodontal therapy; *** statistically significant difference at *p* < 0.001; ** statistically significant difference at *p* < 0.01.

**Table 2:** PD values for sites with moderate (5 mm ≥ PD < 7 mm) and deep (PD ≥ 7 mm) periodontal pockets in the periodontitis group at baseline and at 3-month follow-up after periodontal therapy completion (mean ± SD).

	Periodontitis Group at Baseline	Periodontitis Group after Therapy Completion	*p*-Value
5 mm ≥ PD < 7 mm	5.260 ± 0.225	3.891 ± 0.595	0.000 ***
PD ≥ 7 mm	7.371 ± 0.492	4.967 ± 0.703	0.000 ***

5 mm ≥ PD < 7 mm, moderately deep pockets; PD ≥ 7 mm, deep pockets; *p*-value relates to the difference between the pre- and post-treatment values measured in the periodontitis group; *** statistically significant difference at *p* < 0.001.

**Table 3:** SOD concentration in the periodontitis and the control group at baseline and 3 months after the periodontitis group completed periodontal therapy (mean ± SD).

	Periodontitis Group at Baseline	Control Group	Periodontitis Group after Therapy	^a^ *p*-Value	^b^ *p*-Value	^c^ *p*-Value
Salivary SOD (U/μL)	0.244 ± 0.792	0.017 ± 0.009	0.041 ± 0.052	0.001 **	0.023 *	0.895
Plasma SOD (U/μL)	1.307 ± 0.764	1.112 ± 0.236	1.223 ± 0.629	0.128	0.494	0.386

SOD, superoxide dismutase. ^a^ *p*-value relates to the differences between the periodontitis and the control (periodontitis-free) group at baseline; ^b^ *p*-value relates to the differences between the pre- and post-treatment values obtained in the periodontitis group; ^c^ *p*-value relates to the differences between the control (periodontitis-free) group and the periodontitis group 3 months after the latter completed periodontal therapy; ** statistically significant difference at *p* < 0.01, * statistically significant difference at *p* < 0.05.

**Table 4:** Correlations between the measured clinical parameters and SOD concentration in the saliva and plasma of patients assigned to the periodontitis group at baseline.

	PI	GI	PBI	5 mm ≥ PD < 7 mm	PD ≥ 7 mm	PD (mm)	CAL (mm)
Salivary SOD(U/μL)	0.210	0.149	0.353	−0.323	0.654	0.269	0.333
*p*-value	0.266	0.433	0.056	0.100	0.008 **	0.150	0.072
Plasma SOD(U/μL)	−0.110	−0.227	−0.025	0.052	0.075	0.020	0.077
*p*-value	0.564	0.228	0.896	0.757	0.788	0.916	0.685

PI, plaque index; GI, gingival index; PBI, papilla bleeding index; PD, probing depth; CAL, clinical attachment level; 5 mm ≥ PD < 7 mm, moderately deep pockets; PD ≥ 7 mm, deep pockets; SOD, superoxide dismutase; ** statistically significant difference at *p* < 0.01.

## Data Availability

Not applicable.
